# Intra-Uterine Injections

**Published:** 1874-05

**Authors:** 


*THE MEDICAL EXAMINER.—March* 13, 1874.

Danger of Intra-Uterine Injections—The *Gazette de Joulin* gives the details of two cases, which show that while intra-uterine injections are energetic agents in modifying the conditions of this mucous cavity, they should be employed only with caution.

In one case, though the patient had become enfeebled by repeated hemorrhage, she endured, without suffering inconvenience, two injections of the uterine cavity. A third, consisting of a weak infusion of chamomile and diluted perchloride of iron, was succeeded by death in thirty hours, after decided symptoms of subacute peritonitis. The mucous lining of the uterus and right fallopian tube, and the adjacent peritoneal surface, were found, after death, covered with an ink-black clot, and presenting unmistakable evidences of inflammation.

